# Supplementary material for: Digitized ADOS: Social Interactions beyond the Limits of the Naked Eye
Source: J Pers Med. 2020 Oct 8;10(4):159. doi: 10.3390/jpm10040159 (PMC7711822; doi:10.3390/jpm10040159)
Supplement: Supplementary file 1 [file jpm-10-00159-s001.pdf]

Journal of Personalized Medicine

MDPI

Supplementary Material

https://zenodo.org/record/4029277#.X1-w4WhKiUk

Article

Digitized Autism Observation Diagnostic Schedule: Social Interactions beyond the Limits of the Naked Eye

Harshit Bokadia [1], Richa Rai [1] and Elizabeth Barbara Torres [1,2,*]

[1] Department of Psychology, Rutgers University Center for Cognitive Science, The State University of New Jersey, Rutgers 08854; N.J. USA; hb271@scarletmail.rutgers.edu (H.B.), rr708@psych.rutgers.edu (R.R.)

[2] Department of Computer Science, Computational Biomedicine Imaging and Modelling Centre, Rutgers, The State University of New Jersey, Rutgers 08854; N.J. USA

* Correspondence: ebtorres@psych.rutgers.edu; Tel.: +1-732-208-3158

All Figures match the Figures in the main text but use the angular speed derived from the gyroscopes, instead of the linear acceleration derived from the accelerometers.

Journal of Personalized Medicine

MDPI

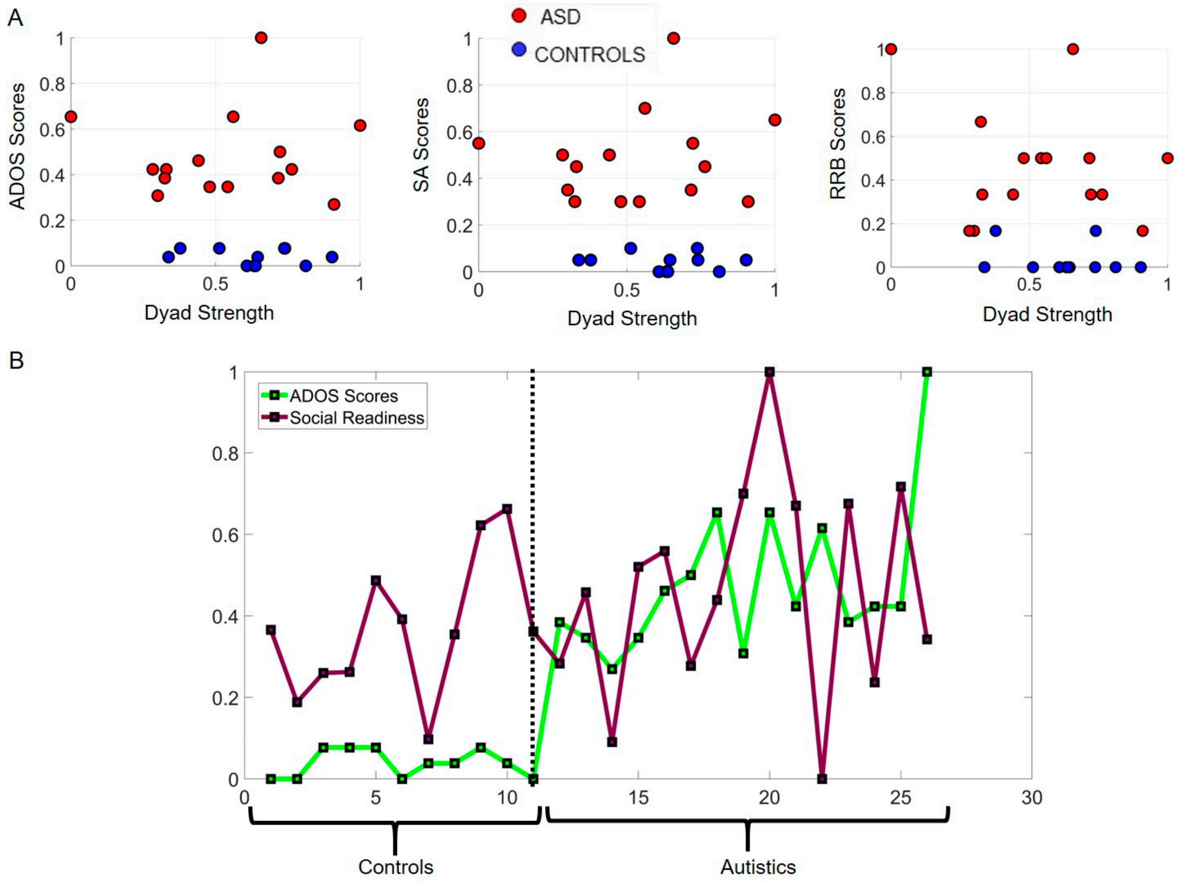

Figure S1. A measure of social readiness potential: Dyadic strength expressed in relation to ADOS sub-scores (Social Affect, SA and the so-called Repetitive Ritualistic Behaviours, RRB). (A) Dyad strength (see methods) tends to be higher in neurotypical controls, with a tendency towards lower (normalized) ADOS scores and some higher variations in the more ambiguous RRB sub-score. The trend in the autistic dyadic strength scores is towards lower values and higher ADOS score, yet 8/15, 53.3% fall within the neurotypical lower range despite higher ADOS scores, thus signaling a hidden capacity for social dyadic exchange. (B) This information can be further unfolded for each participant, whereby a score of social readiness potential is obtained as a relative quantity measuring the difference between each participant and the neurotypical with the highest dyad strength. Many of the autistic participants do have socio-motor strength in the dyadic interaction, despite high ADOS scores.

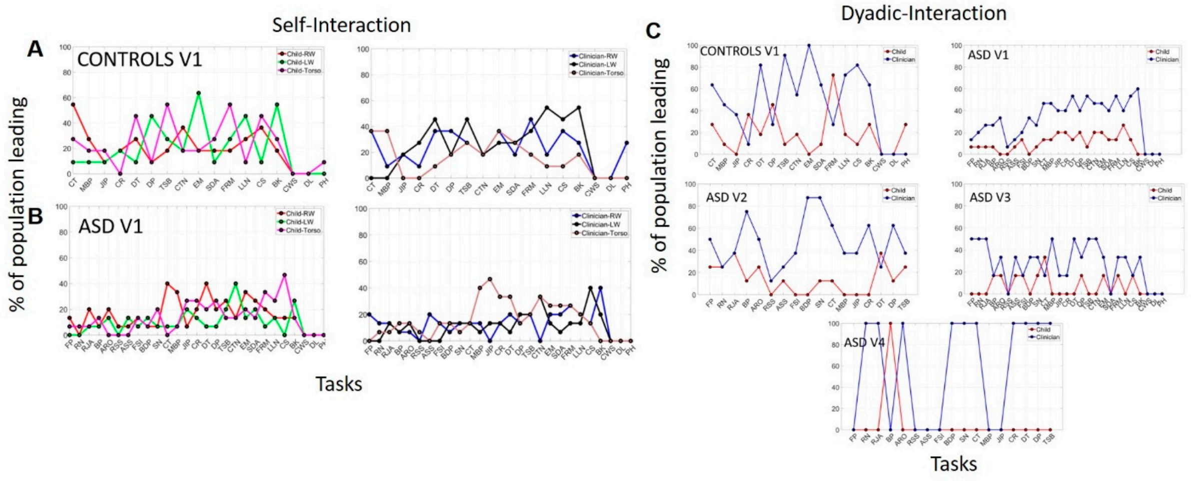

Figure S2. **Mirroring Bias Effects.** (A-B) Biased rating in the ADOS test quantified through mirroring metric of lead-lag social patterns of self-interactions (individual kinematic synergies) quantified as percentage in leading patterns across the cohort. Neurotypical Controls and Clinician show broader range of values than Autistics during the visit one, under most appropriate module and same rater. (C) Mirroring effect and raters' leading bias persist in the shared dyadic cohesiveness as the module and rater change. Visit one and three (appropriate module) under two different raters show a reduction in parameter range for autistics relative to controls. Visit two and four (less appropriate module and by then familiar rater) show an increase in parameter range for autistics relative to visits one and three.

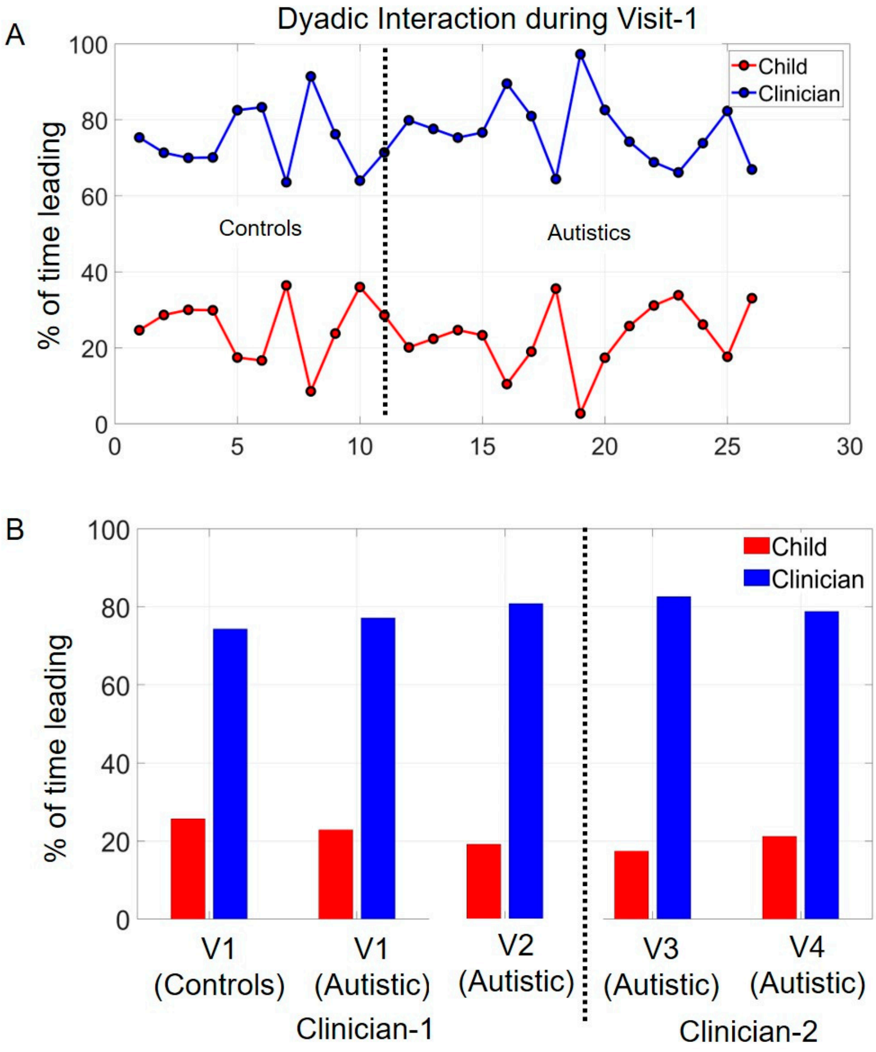

Figure S3. Monologue style of ADOS test. Rater leads social interaction a large percentage of the time for each child. (A) All 26 children divided into neurotypical controls and autistics on the x-axis and on the y-axis, the % of time (taken across all ADOS tasks) that the rater or the child leads the interaction. Clinician rater leads on average for each child, across all tasks. (B) Group data per visit showing the summary of the %time that the person leads the social interaction.

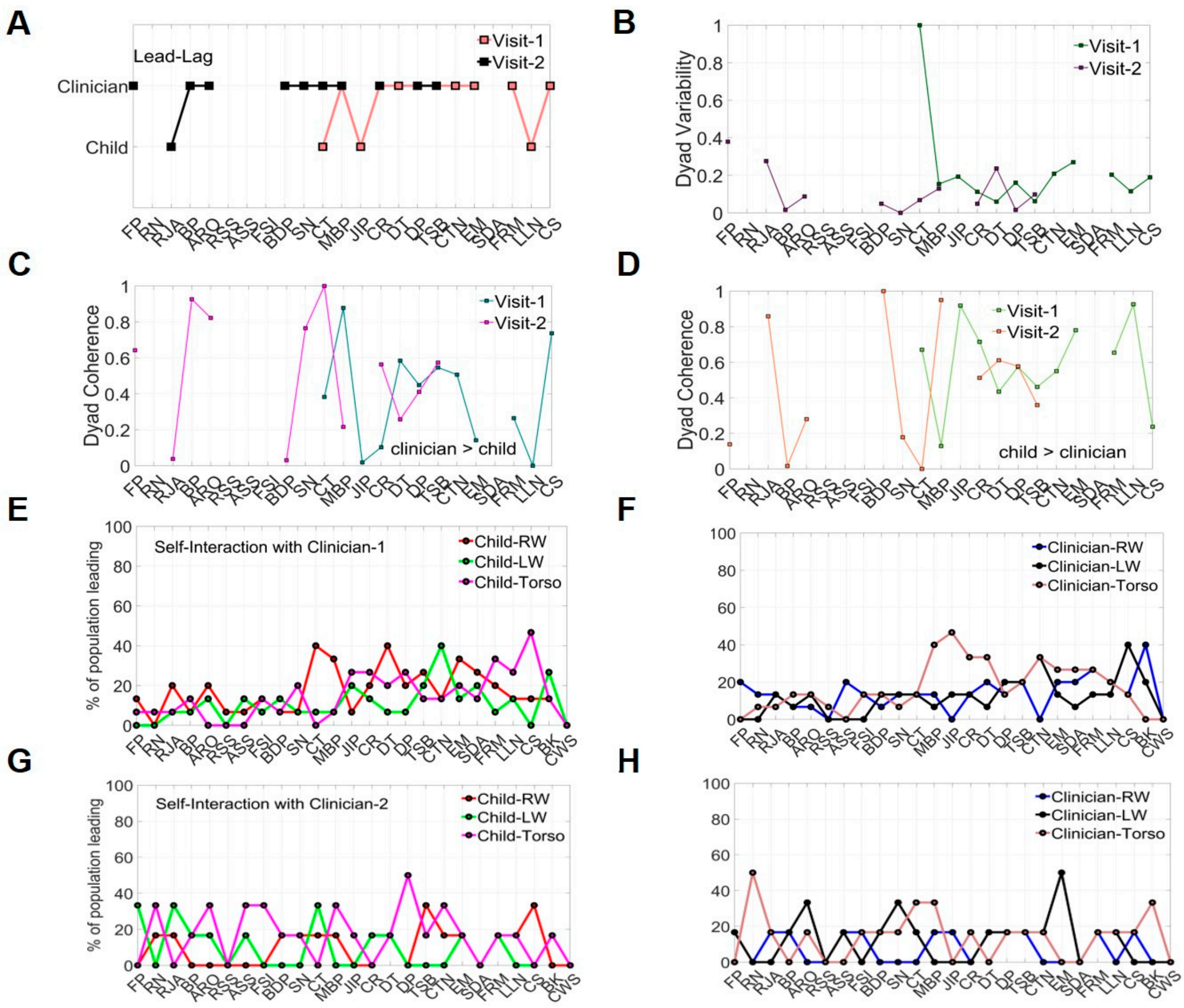

Figure S4. High sensitivity of micro-level metrics captures socio-motor changes over time and serves to measure rater reliability-style. (A-D) ADOS scoring system does not capture change in developmental socio-motor physiology. Across visits, the macro-level scores remain static, but the micro-level socio-motor parameters change. These include leading profile, Dyadic Variability and Dyadic Coherence. (E-F) High sensitivity to changing the clinician rater prevails across all children and tasks. The self-interaction parameters (individual kinematic synergies) of autistic participants are sensitive (at the micro-level) to changing the rater. Leading profiles in the same autistic cohort shift as the raters differ.

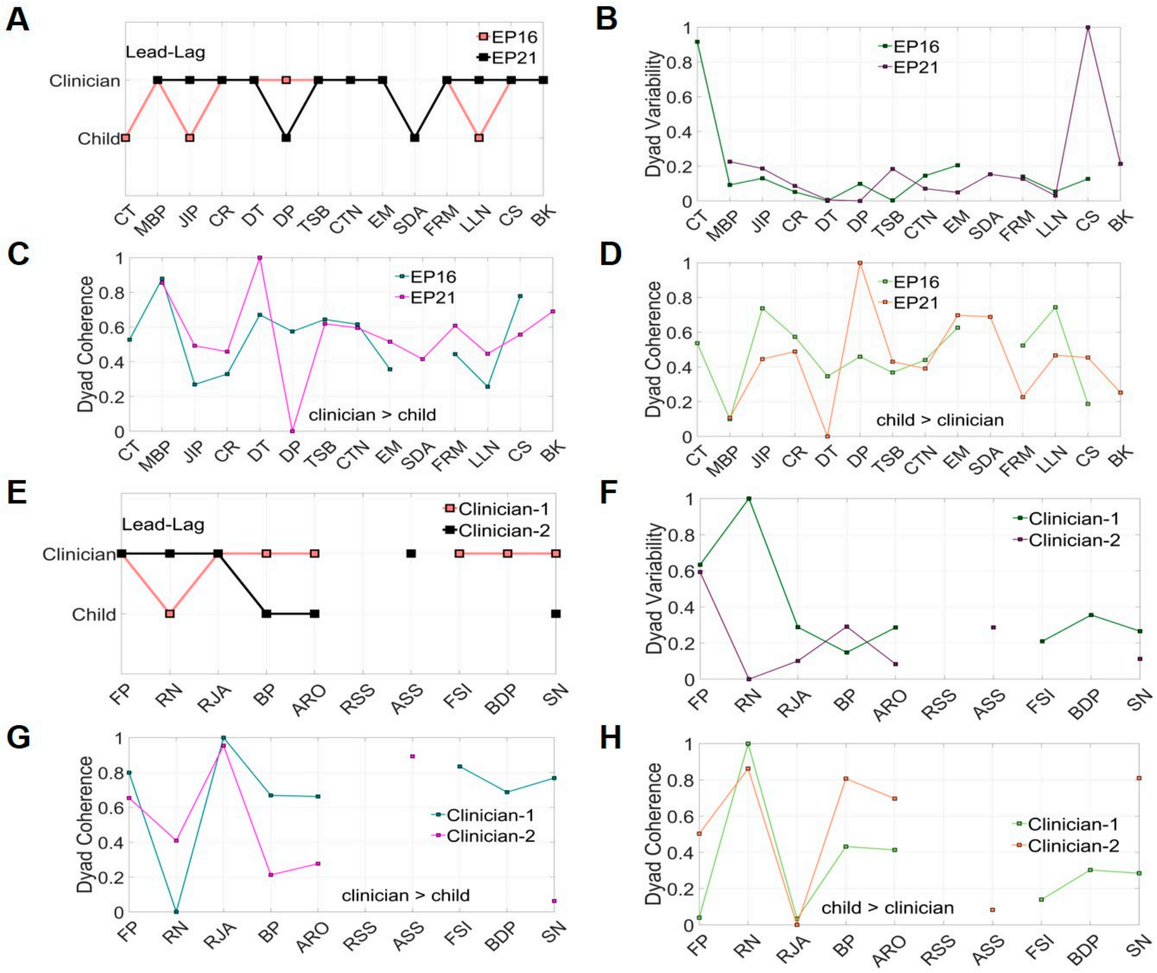

Figure S5. Vignettes samples show bottlenecks of pencil and paper observation methods. (A-D) Macro-level scoring system does not capture change in developmental socio-motor physiology. Experimental participants EP16 and EP21 are perceived very different by the same clinician in relation to leading patterns (A); Dyad Variability (B) and Dyad Coherence (C-D) in each of the clinician→child and child→clinician directions. (E-H) Ill-posed autism detection problem: Given the ADOS score, what is the most likely socio-motor phenotype? Each clinician perceives the same child differently across all micro-level socio-motor indexes.

Journal of
Personalized
Medicine

MDPI

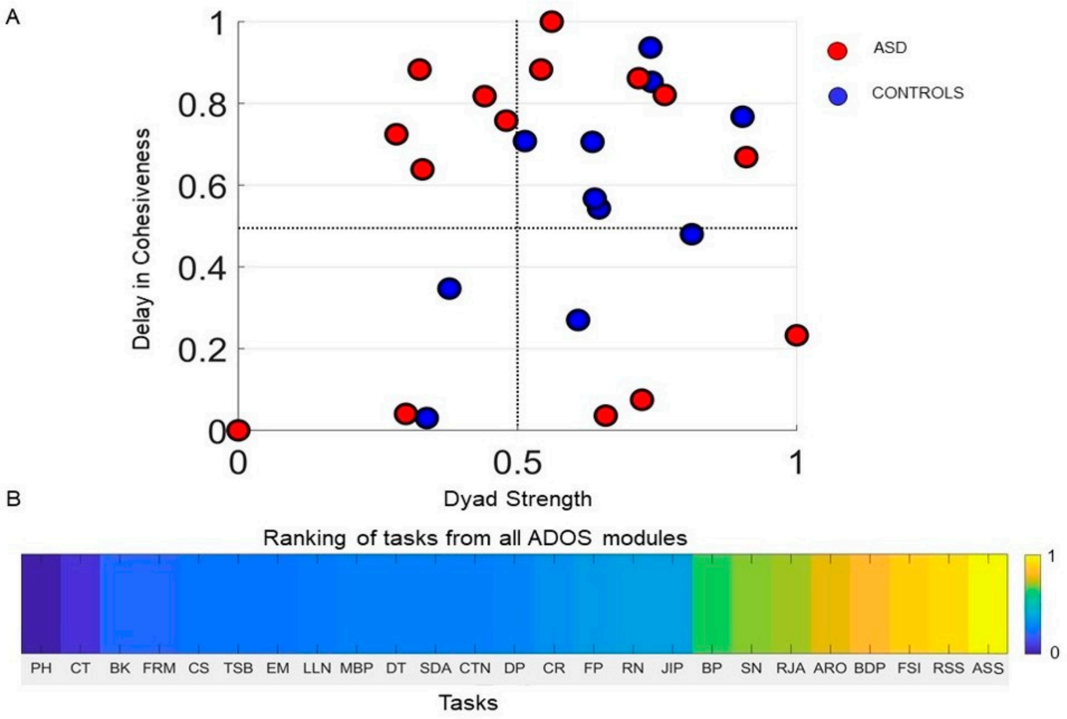

Figure S6. Digital biomarker for personalized design of adaptive targeted therapy. (A) Parameter space spanned by dyad strength on x-axis and delay in cohesiveness (see text for details) on the y-axis. Ideally within a social interaction one would desire high dyad strength and a broad range of response cohesiveness, spanning from fast to slow. Note that most controls have high dyad strength and their responses vary broadly from lower to higher cohesiveness delay, whereas autistics are primarily in the region of low dyad strength and delayed cohesiveness (i.e. they synchronize their body biorhythms with those of the rater, but there is a larger lag than desirable.) (B) Task ranking criteria based on dyad strength calculated for various tasks from participants in visit one. ASS is assigned the highest rank (easiest task to perform) as it depicts the best dyad strength while PH having the lowest dyad strength is assigned the lowest rank (most difficult task to perform).

Table S1: Wilcoxon RankSum test statistics (p-value) at 5% significance level for common tasks between Controls and Autistics.

Task	Child data (Acceleration)	Child data (Angular Velocity)	Child-Clinician data (Acceleration)	Child-Clinician data (Angular Velocity)
CT	0.0562	0.6719	0.2977	0.8760
MBP	0.1835	0.1346	0.4535	0.4272
JIP	0.1035	0.1959	0.4986	0.2358

CR	0.0588	0.4926	**0.0145***	0.6718
DT	0.4288	0.6154	0.9942	0.9669
DP	0.9624	0.5777	0.6413	0.9866
TSB	0.0821	0.0534	0.1678	**0.0035***
CTN	0.4594	0.6198	0.3332	0.8991
EM	**0.0039***	0.6058	0.2102	0.0703
SDA	0.0745	0.9173	0.1430	0.8907
FRM	**0.0236***	0.7568	0.0565	0.2644
LLN	**0.0011***	0.1087	**0.0006***	0.3937
CS	**0.0040***	0.1413	**0.0022***	0.5690
BK	0.4054	0.1811	0.3769	**0.0135***

* denotes that this task has significant differences in NSR values for Autistics and Controls groups.

Table S2: Details of the tasks across all ADOS modules.

Task Number	Abbreviation	Task Name	Module
1	FP	Free Play	1, 2
2	RN	Response to Name	1, 2
3	RJA	Response to Joint Attention	1, 2
4	BP	Bubble Play	1, 2
5	ARO	Anticipation of Routine with Objects	1, 2
6	RSS	Responsive Social Smile	1
7	ASS	Anticipation of Social Routine	1
8	FSI	Functional and Symbolic Imitation	1
9	BDP	Birthday Party	1, 2
10	SN	Snack	1, 2
11	CT	Construction Task	2, 3, 4
12	MBP	Make Believe Play	2, 3

13	JIP	Joint Interactive Play	2, 3
14	CR	Conversation and Reporting	2, 3, 4
15	DT	Demonstration Task	2, 3, 4
16	DP	Description of Picture	2, 3, 4
17	TSB	Telling Story from Book	2, 3, 4
18	CTN	Cartoons	3, 4
19	EM	Emotions	3, 4
20	SDA	Social Difficulties and Annoyance	3, 4
21	FRM	Friends, Relationships and Marriage	3, 4
22	LLN	Loneliness	3, 4
23	CS	Creating a Story	3, 4
24	BK	Break	3, 4
25	CWS	Current Work and School	4
26	DL	Daily Living	4
27	PH	Plan and Hopes	4

Table S3: Details of participants (only whose data is available/ analyzed) across all the visits.

Sr.NO	Participant ID	Age	Sex	Visit-1 Module (Total Score)	Visit-2 Module (Total Score)	Visit-3 Module (Total Score)	Visit-4 Module (Total Score)
1	CP01	8	F	3 (0)	-	-	-
2	CP02	10	F	3 (0)	-	-	-
3	CP03	9	F	3 (2)	-	-	-
4	CP04	12	F	3 (2)	-	-	-
5	CP06	7	M	3 (2)	-	-	-
6	CP08	9	F	3 (0)	-	-	-
7	CP09	7	F	3 (1)	-	-	-
8	CP10	11	M	3 (1)	-	-	-
9	CP11	15	M	4 (2)	-	-	-
10	CP12	11	F	4 (1)	-	-	-
11	CP13	13	M	4 (0)	-	-	-
12	EP01	4	M	3 (10)	2 (7)	3 (9)	2 (8) *X
13	EP02	8	M	3 (9)	2 (8) *X	-	-

14	EP03	10	M	3 (17) *X	2 (13)	3 (24) *X	2 (21) *X
15	EP04	13	F	3 (7)	4 (8) *X	3 (11) *X	4 (8) *X
16	EP05	6	M	3 (9)	2 (7)	3 (8) *X	-
17	EP07	11	M	3 (12)	2 (9) *X	3 (18) *X	2 (13) *X
18	EP09	5	M	1 (18) *X	1 (16)	-	-
19	EP10	9	M	1 (13)	1 (17)	-	-
20	EP13	6	M	3 (17)	-	-	-
21	EP14	14	M	1 (8)	2 (10)	1 (14)	2 (15) *X
22	EP15	10	M	1 (17)	1 (15) *X	1 (26)	1 (21) *X
23	EP16	4	M	3 (11)	2 (11)	3 (22) *X	2 (20) *X
24	EP17	11	M	1 (16)	1 (18) *X	1 (18)	1 (19) *X
25	EP18	9	F	3 (10)	2 (11)	3 (16)	2 (10)
26	EP19	7	M	3 (8) *X	2 (8) *X	3 (8)	-
27	EP20	7	M	3 (11)	2 (11) *X	3 (17) *X	2 (16) *X
28	EP21	11	F	3 (11)	2 (9) *X	3 (8) *X	2 (11) *X
29	EP22	4	M	1 (26)	1 (24) *X	-	-

*X denotes that the subject came for the visit, but his/her data is not analyzed either because the dataset was not available/lost/corrupted or information available was not enough to do the analysis.
